# Assessment of TSLP, IL 25 and IL 33 in patients with shrimp allergy

**DOI:** 10.1186/s13223-021-00576-9

**Published:** 2021-07-23

**Authors:** Natalia Ukleja-Sokołowska, Magdalena Żbikowska-Gotz, Kinga Lis, Rafał Adamczak, Zbigniew Bartuzi

**Affiliations:** 1 Department of Allergology, Clinical Immunology and Internal Medicine, Ludwik Rydygier Collegium Medicum in Bydgoszcz, Nicolaus Copernicus University in Toruń, ul. Ujejskiego 75, 85-168 Bydgoszcz, Poland; 2 Department of Obstetrics and Gynecology, Ludwik Rydygier Collegium Medicum in Bydgoszcz, Nicolaus Copernicus University in Toruń, Bydgoszcz, Poland

**Keywords:** Shrimp allergy, sIgE, TSLP, IL-25, IL-33, ImmunoCap ISAC, House dust mite, Pen m 1, Der p 1, Der p 2

## Abstract

**Background:**

Shrimp allergy is a growing problem among the European population. TSLP, IL-25 and IL-33 are involved in the pathophysiology of allergic diseases, including asthma and atopic dermatitis, as they activate the Th2-dependent immune response.

**Methods:**

Thirty-seven patients (18 male and 19 female) with a positive history of symptoms associated with shrimp consumption were selected. All patients had blood samples taken to assess the concentration of allergen-specific IgE (sIgE) to house dust mites (HDM) and shrimp (Singleplex, quantitative method with cut off value > 0,35 kAU/L) as well as the level of allergen components using the ImmunoCap ISAC method (Microarray test, semi-quantitative with cut off value > 0,3 ISU-E). The concentrations of TSLP, IL-25 and IL-33 in the patients’ blood serum was assessed using the ELISA method (Cusabio). Twenty patients with negative allergy history of allergic disease tests were included in the control group.

**Results:**

Among the 37 shrimp-allergic patients, ImmunoCap ISAC was identified the presence of sIgE to the available shrimp allergen components in only 14 cases (37.8%). TSLP and IL25 levels were significantly higher in the study group. No statistically significant correlation was found between the concentration of analyzed alarmins and the concentration of sIgE level to shrimp or HDM between the study and control groups. No statistically significant correlation was found between poly-sensitization occurring in patients and levels of TSLP, IL-25 and IL-33 .

**Conclusion:**

In shrimp-allergic patients, the concentrations of TSLP and IL-25 were significantly higher than in the control group (1.33 vs. 0.49 and 157 vs. 39.36, respectively). There was no correlation between the concentrations of TSLP, IL-25 and IL-33 and the concentration of sIgE in the patients or the number of allergen components that the patients were sensitized to.

*Trial registration:* Bioethics Committee 147/2015, 11.03.2015.

**Supplementary Information:**

The online version contains supplementary material available at 10.1186/s13223-021-00576-9.

## Background

The pathogenesis of allergic diseases remains the subject of considerable interest in the field of immunology. Many reports emphasizing the important role of the so-called alarmins (*damage-associated molecular patterns, DAMP*) i.e., TSLP, IL-25 and IL-33, have been published recently. These cytokines are of epithelial origin and are triggered by, among others, damaging factors, viral infections and allergic inflammation [1]. They are also involved in the pathophysiology of allergic diseases, including asthma and atopic dermatitis, as they activate the Th2-dependent immune response [2].

The role of TSLP is extremely important in the development of allergic inflammation in the airways. In 2005, Zhou B et al. proved that mice lacking TSLP did not develop inflammation of allergic etiology in the respiratory tract [3].

An interesting report was published in 2009 by Headley M et al. The researchers evaluated the respiratory response of mice to TSLP, administered alone or in combination with ovalbumin (OVA), every other day for two weeks. It was found that the mice treated concomitantly with TSLP and OVA showed significantly more pronounced airway inflammation and eosinophilia compared to mice receiving TSLP alone. These results indicate that allergen exposure and TSLP interaction play the important role of the antigen in the development of inflammation of allergic etiology [4].

Hui C. et al. studied the effect of TSLP in vitro in ten patients with atopy and ten atopy-free subjects. In this case, atopy was defined as positive skin tests for at least one allergen in a standard set of 14 inhalant allergens. Subsequently, CD 34 + progenitor cells were isolated from peripheral blood and incubated with IL-3 (1 ng/mL), TSLP (10 ng/mL) and anti-TNFα (10 µg/mL). In the presence of IL-3, TSLP was found to significantly promote the formation of eosinophilic/basophil colony-forming units from the human progenitor cells. Moreover, IL-3/TSLP-stimulated progenitor cells actively secreted a series of cytokines/chemokines. Among this series, TNFα was assigned a key role, which increased the surface expression of TSLPR in the presence of IL-3. Particularly interesting was the finding that progenitor cells isolated from atopic individuals were functionally and phenotypically more sensitive to TSLP than those obtained from non-atopic individuals [5].

In a study published in 2012, Han H et al. showed evidence that TSLP also played a role in the development of allergic diseases in a patient. The phenomenon of acquiring further allergic diseases, commonly known as allergic march, has a complex and not fully explained etiology. The researchers sensitized mice using OVA and TSLP via the transdermal route (alone or in combination), resulting in the development of atopic dermatitis. After a nine-day break, the mice were challenged with OVA intranasally, and the development of inflammation in the airways was observed. The researchers confirmed that the mice’s exposure to transdermal TSLP without coexistent antigen did not cause inflammation in the airways, while in mice that were sensitized with OVA alone, the allergy did not extend to the respiratory tract. This indirectly proves that TSLP is one of the factors playing a key role in the allergic march [6].

In 2013, Imai Y et al. published a highly relevant study that evaluated transgenic mice modified to express IL-33 in the epidermis. Despite normal development during the first weeks of life, at six to eight weeks of age, the mice developed dermatitis. This development occurred due to the phenotype of atopic dermatitis, which resulted in severe itching, despite the absence of pathogens in the environment. An increase in IL-5 and IL-13 expression, RANTES/CCL5 activity and secretion as well as eotaxin 1/CCL11 was observed in the blood serum, whereas IL-33 had no effect on TNF-α, IFN-γ and TSLP [7].

Han H et al. studied the function of IL-33 and the relationship between IL-33 and TSLP in case of gastrointestinal allergic symptoms. The tests were performed on mice that were sensitized to OVA. IL-33 was found to promote the development of inflammation of allergic etiology independent of TSLP, while the absence of IL-33 in the presence of TSLP protected the mice against diarrhea after exposure to the allergenic protein. Moreover, the blockade of IL-22 in sensitized mice suppressed pathological symptoms, including those affecting the skin. These results indicate that IL-33 plays a critical role in both the development of the early phase of dermatitis as well as subsequent gastrointestinal symptoms, indicating the important role of IL-33 in the allergic march [8].

The current study is focused on the level of alarmin in food allergic patients, based on a cohort of shrimp-sensitized adult patients. Shrimp allergy is a growing problem among the European population. There are large geographical differences in the prevalence of food allergy across Europe. In a EuroPrevall study in school children, the prevalence of declared symptoms of shrimp sensitization differed between different parts of Europe and was found to be between 0.38 and 3.75% [9].

Symptoms associated with shrimp consumption can fall within a wide spectrum, from mild local reactions to generalized reactions, including anaphylactic shock.

One of the most interesting issues related to the shrimp allergy is that the main allergen of the crustaceans is tropomyosin, which has a high interspecies homology. It is also found in house dust mites. Other shrimp allergens have also been reported, which may also be associated with a severe course of sensitization [10].

Component-resolved diagnosis allows us to learn about the patient’s allergic profile. Analysis of the data acquired for patients who are sensitized to shrimp allergens may enable us to evaluate the relationship between the allergy and a specific allergen component as well as the clinical course of the disease; it can also help us enable the isolation of high-risk patients and identify potential sources of cross-reactivity in patients.

The aim of this research is to establish the concentrations of TSLP, IL-33 and Il-25 in patients who are sensitized to shrimp. These cytokines play a crucial role in activating the Th2-dependent immune response. This immune response can help us evaluate if their level influences the clinical course of food allergy. To our knowledge, this is the first study that discusses the role of alarmins in shrimp allergy.

## Methods

Thirty-seven patients (18 male and 19 female) with a positive history of symptoms associated with shrimp consumption were selected from patients of the Ward and Outpatient Clinic of Allergic Diseases in the Department of Allergology, Clinical Immunology and Internal Medicine, Collegium Medicum, Nicolaus Copernicus University in Bydgoszcz, Poland.

The positive history of symptoms associated with shrimp was defined as a reaction that occurred within minutes to hours of shrimp ingestion in the 12 months before the research was conducted. The symptoms include urticaria, edema of lips and tongue, dyspnea and cough, allergic rhinitis and conjunctivitis and anaphylaxis. The source of symptoms was confirmed in all cases by positive skin prick test with a frozen shrimp purchased from a local eco-shop.

Patients being treated for serious, chronic diseases or were on medication that could influence the results of this study were not included in the research (For detailed exclusion criteria, please see Additional file 1).

A detailed allergology interview and physical examination were conducted for each patient. All patients had a skin prick test with house dust mite (*Dermatophagoides pteronyssinus and Dermatophagoides farinae*) extracts using the Allergopharma set, as well as prick-by-prick tests with tiger shrimp bought from local eco-market. As per the current European standard, the tests were considered positive when the diameter of the wheal of each particular test was ≥ 3 mm.

The blood samples of all patients were taken to assess the concentration of allergen specific IgE to house dust mite (*Dermatophagoides pteronyssinus and Dermatophagoides farinae* allergen extract) and shrimp (*Pandalus borealis, Penaeus monodon, Metapenaeopsis barbata, Metapenaeus joyner* extract).

The study was conducted using venous blood serum. Blood was taken in accordance with the standard conditions, between 7:00 am and 9:00 am. The patients were fasting after an overnight rest. Blood was collected from the median cubital vein using a closed vacuum system (Vacuette, Greiner Bio-One), into a ‘CAT Serum Sep Clot Activator’ 5 ml tubes. The blood samples, after being left to finish the clotting mechanism, were centrifuged for 15 min. at 3500 rotations per min. The serum was immediately separated and kept frozen at -70 °C until it was assayed.

All immunological determinations were performed with the use of the highly sensitive, immune-fluorescent ImmunoCap method (Thermo Fisher Scientific). The concentrations of sIgE were found to increase when they exceeded 0.35 kUA/l (ImmunoCap) in accordance with the common practice in the field. In all patients, the level of allergen components was determined using ImmunoCap ISAC method. The level of sIgE in ImmunoCap ISAC was found to increase when it exceeded 0.3 ISU-E (ISAC Standardized Units).

The concentrations of TSLP (0.625 ng/ml-40 ng/ml), IL-25 (62.5 pg/ml–4000 pg/ml) and IL-33 (15.6 pg/ml-1000 pg/ml) in blood serum of the patients were assessed using the ELISA method (Cusabio).

Twenty patients (12 female and 8 male, aged 29–61, average age 43.4) with a negative allergy history of allergic disease tests were included in the control group (CG).

### Statistical analysis

Mann–Whitney and Kruskal–Wallis tests, with Dunn test post hoc analysis, were conducted, where applicable. The analyses were prepared using the R program, version 3.3.1 and MS Excel 365.

The study was approved by the local Bioethical Committee and was assigned a classification number: 147/2015. All patients gave informed, written consent to participate in the study.

## Results

The characteristics of the study population is given in the Table 1. No significant differences between the study and control group in terms of age and sex were found.

**Table 1:** General characteristics of the study group and control

Parameter		Group		*p*
		Study group (N = 37)	Control group (N = 20)	
Age	Mean ± SD	42.14 ± 13.93	43.4 ± 10.27	*p* = 0.644
	Median	39.5	45	
	Quartiles	33.75–49.25	33–49.25	
Sex	Women	19 (51.35%)	12 (60.00%)	*p* = 0.729
	Men	18 (48.65%)	8 (40.00%)	

*p* for quantitative variables Mann–Whitney test, for qualitative variables the Chi-squared test or exact Fisher test

The number of patients with elevated concentration of specific IgE (≥0,35 kUA/l) are presented in Table 2. All patients in the group had elevated level of specific IgE to shrimp, which was the inclusion criteria.

**Table 2:** Specific IgE concentration was determined by the ImmunoCap method and the level of IgE against selected allergen components was determined by the ImmunoCap ISAC method

Results of ImmunoCap assay			
	Number of patients with elevated concentration (≥0.35 kUA/l) n = 37	Range	Avg. of elevated results
IgE shrimp (*Pandalus borealis, Penaeus monodon, Metapenaeopsis barbata, Metapenaeus joyner* extract)	37	0.69–100	10.4
IgE *Dermatophagoides pteronyssinus*	29	0.42–100	35.1
IgE *Dermatophagoide farinae*	25	0.39–100	38.6

Results of ImmunoCap ISAC immunoassay (incl. components of HDM and shrimp + the most frequently sensitizing allergen components in the group)			
IgE	Number of patients with elevated level (≥0.3 ISU-E). n = 37	Range ISE-U	Avg. of elevated results
Der p 1	18	0.4–94	21.4
Der p 2	23	0.7–100	37.9
Der p 10	11	0.4–100	24.8
Der f 1	18	0.5–47	13.3
Der f 2	23	0.4–100	26.3
Pen m 1	8	1.1–100	29.1
Pen m 2	6	0.6–68	22.5
Pen m 4	2	7.65–4.3	7.7
Fel d 1	16	0.4–100	20.9
Cyn d 1	15	0.4–13	3.4
Phl p 1	14	0.4–29	10.8
Bet v 1	13	0.4–100	32.1
Phl p 4	12	0.4–14	3.9
Bla g 7	10	0.5–100	21.9
Cor a 1	9	1.6–35	14.8
Mal d 1	9	5.1–62	19
Aln g 1	8	0.6–62	14.8
Ani s 3	8	1.2–100	23.7
Ara h 8	8	0.5–35	8.2
Art v 1	8	0.3–26	5.5
Blo t 5	8	0.4–24	7.0
Can f 1	8	0.3–48	19.3
Can f 5	8	0.5–72	24.9
Lep d 2	8	0.5–100	27.9
Pru p 1	8	0.8–0.8	7.1
Gly m 4	7	0.4–8.3	4.2
Ves v 5	7	0.5–5.3	1.8

It was found that in the study group, among patients who had clinical symptoms associated with consumption of shrimps, allergy to *Dermatophagoides pteronyssinus* occurred in as many as 29 patients (78.4%). Elevated levels of sIgE to the main shrimp allergen, Tropomyosin Pen a 1, occurred only in seven patients in this group (18.9%).

**Fig. 1:**
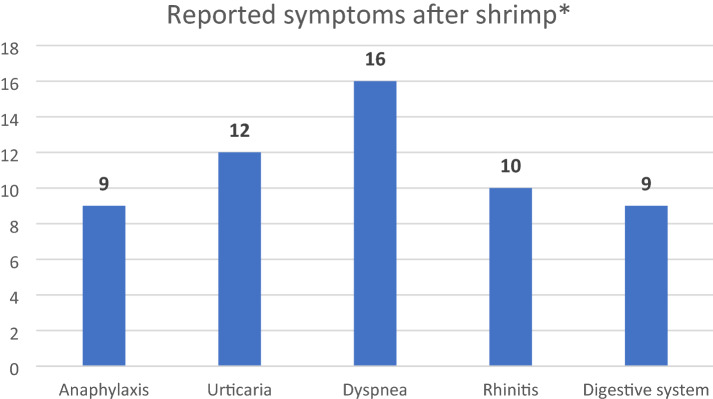
Clinical symptoms declared by patients. *Anaphylaxis was diagnosed based on EAACI criteria. Symptoms from digestive system were associated with stomach cramps and pain or diarrhea and vomiting. Rhinitis symptoms were associated in half of cases with allergic conjunctivitis

The results of the ImmunoCap ISAC test in patients are notable. Among the three shrimp allergen components available in this micro-array test, elevated sIgE was found for Pen m 1 in eight cases, Pen m 2 in six cases and Pen m 4 in two cases (Table 2). It is worth noting that 12 patients were sensitized to only one shrimp allergen component, while two patients were sensitized to two allergen components. Overall, among the 37 patients with a history of self-declared shrimp-related allergy symptoms post consumption and elevated shrimp-specific IgE (ImmunoCap), ImmunoCap ISAC identified the presence of sIgE in at least one of the available shrimp allergen components in only 14 cases (37.8%). Some patients were sensitized to more than one allergen component at the same time. The clinical symptoms declared by the patients are presented in Fig. 1.

Simultaneously, the allergen profile of patients with shrimp allergy was noteworthy. Sensitization to dust mite allergen components dominated, but as many as 16 patients had elevated levels of sIgE to Fel d 1. Sensitization to airborne allergens of grass pollen (Cyn d 1, Phl p 1, Phl p 4) and trees (Bet v 1) was also common. It should also be noted that elevated levels of sIgE to Bla g 7, cockroach tropomyosin, were found in as many as ten patients. The allergen profile of the study group is presented in Table 2.

In the control group, there were no elevated levels of sIgE to shrimp and house dust mites.

**Fig. 2:**
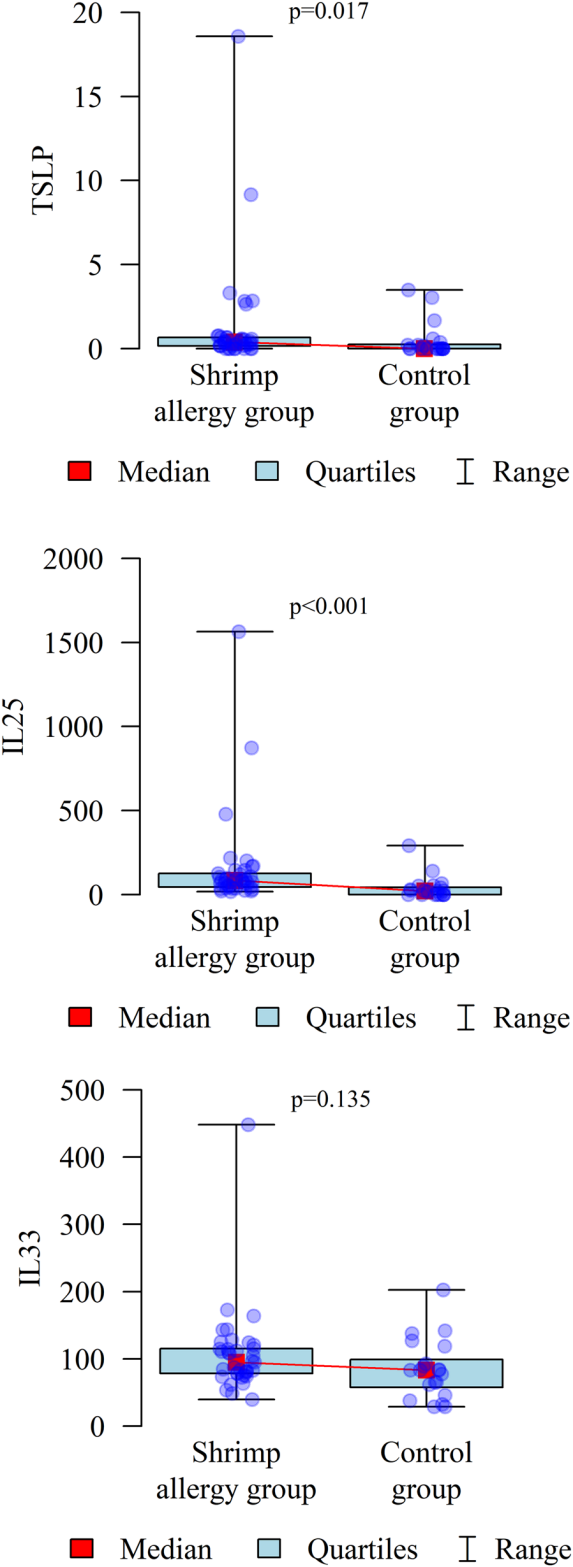
Levels of TSLP, IL 33 and IL 25 in the study group and in the control group

Levels of TSLP, IL-33 and IL-25 were determined in the study group and in the control group. It was found that TSLP and IL25 levels were significantly higher in the study group compared to the control group (*p* < 0.05). The results of the analysis are presented in Table 3 and Fig. 2.

**Table 3:** Levels of TSLP, IL 33 and IL 25 in the study group and in the control group

Parameter		Group		*p*
		Study group (N = 37)	Control group (N = 20)	
TSLP (ng/ml)	Mean ± SD	1.33 ± 3.34	0.49 ± 1.03	*p* = 0.017*
	Median	0.39	0	
	Quartiles	0.16–0.67	0–0.25	
IL25 (pg/ml)	Mean ± SD	157.18 ± 281.77	39.36 ± 68.48	*p* < 0.001*
	Median	84.02	22.3	
	Quartiles	45.72–125.72	0–44.15	
IL33 (pg/ml)	Mean ± SD	106.76 ± 65.25	84.32 ± 44.15	*p* = 0.135
	Median	94.99	83.17	
	Quartiles	78.3–115.28	57.61–99.08	

*p* Mann–Whitney test

*Statistically significant correlation (*p* < 0.05)

No statistically significant effect of age on TSLP, IL-25 and IL-33 levels was found in the study group. Moreover, while it was shown that TSLP concentration was significantly higher in women in the study group (*p* < 0.05), no such relationship was found in the control group.

**Fig. 3:**
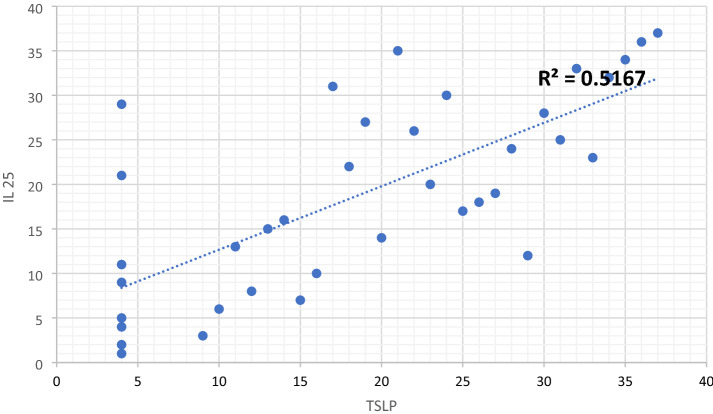
TSLP and IL 25 levels (Spearman's correlation)

The TSLP concentration in the study group correlates positively and significantly with IL-25 (*p* < 0.05), while no similar relationships were found for TSLP and IL-33 and between IL-33 and IL-25 (Fig. 3).

Relationships between the concentration of sIgE to shrimp, D. pteronyssinus and D. farinae and the concentrations of TSLP, IL-25 and IL-33 were analyzed. No statistically significant correlation was found between levels of analyzed alarmins and the sIgE levels (Table 4).

**Table 4:** A correlation between sex and TSLP, IL25 and IL 33 levels in the study population of patients allergic to shrimp allergens

Parameter		Sex		*p*
		Women (N = 19)	Men (N = 18)	
TSLP (ng/ml)	Mean ± SD	2.11 ± 4.51	0.5 ± 0.84	*p* = 0.036*
	Median	0.57	0.24	
	Quartiles	0.25–0.77	0.04–0.44	
IL25 (pg/ml)	Mean ± SD	212.56 ± 375.55	98.73 ± 108.4	*p* = 0.159
	Median	93.35	68.93	
	Quartiles	66.55–135.86	38.33–104.15	
IL33 (pg/ml)	Mean ± SD	101.42 ± 25.31	112.4 ± 90.95	*p* = 0.558
	Median	107.95	85.94	
	Quartiles	80.47–114.94	74.51–121.22	

*p* Mann–Whitney test

*Statistically significant correlation (*p* < 0.05)

**Fig. 4:**
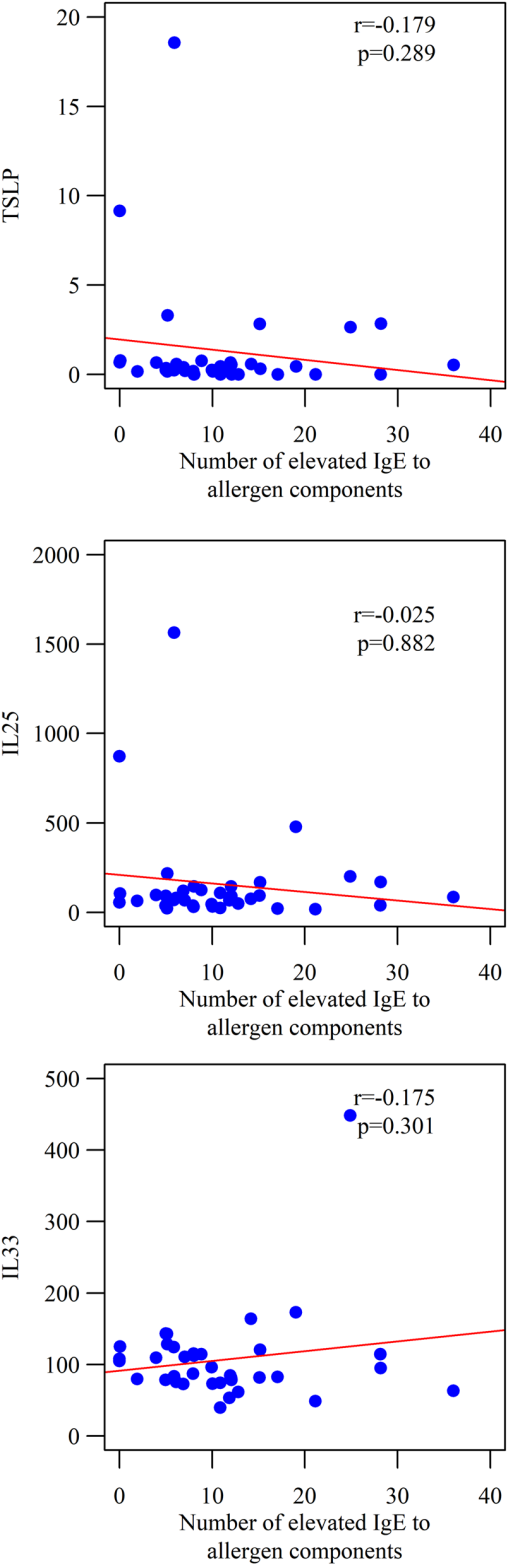
The number of positive allergen components in a patient in the ImmunoCap ISAC test and the concentration of TSLP, IL 25 and IL 33

Recognizing the significant effect of TSLP, IL-25 and IL-33 on the pathogenesis and development of allergic diseases and the effect of elevated concentrations of these alarmins on the number of allergen components to which the patient was sensitized was analyzed in the ImmunoCap ISAC test. No statistically significant correlation was found between poly-sensitization occurring in patients and levels of TSLP, IL-25 and IL-33 (Fig. 4).

There was also no relationship between the concentrations of TSLP, IL-25 and IL-33 and the nature of the clinical symptoms reported by patients and their severity.

## Discussion

The role of IL-25, IL-33 and TSLP in various allergic diseases has been studied for several years. Identifying the relationship between these cytokines and the natural history of these diseases gives hope for a breakthrough in the prognosis and the possibility of utilizing new biological drugs for specific diseases.

To the best of our knowledge, no study has been published that analyzes TSLP, IL25 and IL-33 in a group of adult patients with food allergy symptoms related to shrimp consumption. To date, only one paper has been published in this context, in which the authors report a pathway of immune counter-regulation that suppresses the development of aeroallergy and shrimp-induced anaphylaxis in a mice model. The authors found that signaling through epithelial expressed dectin-1 suppresses the development of type 2 immune responses through inhibition of interleukin-33 secretion and the subsequent recruitment of IL-13-producing innate lymphoid cells [11].

In our study, 37 allergy patients diagnosed with an allergy to shrimp were considered. The condition was diagnosed on the basis of the patient’s clinical symptoms after shrimp consumption and elevated shrimp-specific IgE levels by the ImmunoCap method. Compared to the control patients, elevated TSLP, IL-33 and IL-25 levels were found. Similar conclusions were drawn by Ying S et al., who compared cell TSLP-dependent chemokine expression in patients with bronchial asthma and COPD. The researchers found that the expression of mRNA-encoding TSLP, TARC/CCL17, MDC/CCL22 and IP-10/CXCL10, other than I-TAC/CXCL11 and I-309/CCL1, were significantly increased in severe asthma and COPD patients compared to the non-smoking control group. Similar findings were made in the examination of fluid collected during bronchoalveolar lavage. The expression of analogous chemokines was found in current and former smokers. Increased TSLP expression in bronchial mucosa was found in patients with bronchial asthma and COPD [12]. Tang et al. evaluated the expression of IL-25, IL-17RA and IL-17RB as well as serum IL-25 levels in 14 patients with atopic asthma, 15 patients with only atopy and 14 healthy people in the control group. The expression of IL-17RB and IL-17RA on eosinophils were found to be significantly higher in patients with atopic asthma. Serum IL-25 concentration was higher in people with atopy compared to healthy people [13]. These results are consistent with our findings.

It is worth emphasizing that in our studies, no relationship was found between the concentrations of TSLP, IL-25 and IL-33 and the nature and severity of clinical symptoms after eating shrimp, declared by the patient. In an interesting publication from 2016, Nygaard U et al. examined the concentrations of TSLP, IL-31, IL-33 and soluble ST2 in adults and children with atopic dermatitis, compared to the control group. They found that the concentrations of TSLP, IL-31 and IL-33 correlated with each other and were higher in patients with AD compared to the control group. However, no correlation was found between the concentrations of these cytokines and severity of clinical symptoms [14]. This absence of correlation between the symptoms of TSLP and IL-33 levels is consistent with our observations. In our study, however, TSLP concentration correlated with the level of IL-25, but not IL-33. On the contrary, in 2013, Sano Y. et al. published results of an interesting study, in which they analyzed TSLP in the stratum corneum (scTSLP) in AD patients. They found that scTSLP expression was increased in patients with AD, compared to the healthy population. In addition, a correlation was found with the severity of skin lesions on the SCORAD scale, particularly while assessing the severity of dry skin [15].

Additionally, Noti M. et al. found that sensitization to food allergens through an atopic dermatitis-like skin lesion is associated with an expansion of TSLP-elicited basophils in the skin, which promote antigen-specific Th2 cytokine responses, elevated antigen-specific serum IgE levels and the accumulation of mast cells in the intestine, promoting the development of intestinal food allergy [16].

ImmunoCap ISAC tests showed that in the group of patients who were allergic to shrimp, allergy to house dust mites predominated. 18 and 23 patients, respectively, were allergic to Der p 1 and Der 2 (48.6 and 62% of the study population). Elevated sIgE levels to the allergenic extract of Dermatophagoides pteronyssinus and Dermatophagoides farinae occurred in 29 and 25 patients (78.4 and 67.6%), respectively. Boquete et al. indicated that 71% of the patients who were allergic to house dust mites also had sIgE to shrimp, and 55% of them had increased level of IgE specific to shrimp tropomyosin [17]. Canadian studies demonstrated a high incidence of allergy to house dust mites in 95 patients with a confirmed allergy to shrimp. In that study population, 86 (90.5%) patients had positive skin prick tests for house dust mite allergens [18]. As our previous observations indicated, among 232 patients with symptoms of allergic rhinitis and as many as 59 patients had elevated levels of shrimp sIgE, while only 12 were not allergic to house dust mites (20.3%) [19]. The differences may be due to a different population selection criterion, however, the high frequency of mite allergy in patients allergic to shrimp allergens is confirmed.

The analysis of other allergen components, which patients with a shrimp allergy are allergic to, is also interesting. We would expect that allergen components available in the ImmunoCap ISAC will be present in a significant number of patients. Unfortunately, the limitation was the fact that only three components (Pen m 1, Pen m 2, Pen m 4) were available in this study:

- *Pen m 1, or tropomyosin* is the main allergen of shrimps, a muscle protein that plays an important role in the muscle contraction process [20]. Tropomyosin is believed to be the main cause of symptoms developing after ingestion of shrimps, as well as the main cause of a cross-allergy with house dust mites, and other allergens [21].
- *Pen m 2, or arginine kinase* is a protein that is identified in several varieties of shrimp, such as in *Litopenaeus vannamei* (Lit v 2) and *Penaeus monodon* (Pen m 2). Studies conducted in Italy have demonstrated that it is a less potent allergen causing hypersensitivity in 10–15% of the population of patients allergic to shrimp [10].
- *Pen m 4, or Sarcoplasmic calcium binding protein* is a muscle protein that plays an important role in the process of muscle contraction. It is similar to arginine kinase, as it is a less potent allergen. However, it causes hypersensitivity in approximately 10–15% of shrimp-sensitized patients, which is clinically significant [10].

Overall, among 37 patients with a history of shrimp-related symptoms and elevated shrimp-specific IgE (ImmunoCap), ImmunoCap ISAC identified the presence of sIgE to available shrimp allergen components in only 14 cases (37.8%). This indicates that although component diagnostics based on the ImmunoCap ISAC test is important in assessing the allergen profile of patients, in the case of shrimp allergy, it plays an auxiliary role, and a negative result in the context of the allergen components Pen m 1, Pen m 2 and Pen m 4 does not rule out the shrimp allergy. In our previous research, we found that, in case of shrimp sensitization, the results of sIgE levels against corresponding allergen components determined using singleplex ImmunoCap (quantitative and highly sensitive) and ImmunoCap ISAC (semi-quantitative, multiplex micro-assay test) are similar in most cases, with correlations above 0.7 [22].

There are many other proteins contained in shrimp meat that can cause allergic symptoms in patients. In 2012, Asero et al. found that only 41% of patients had an elevated level of sIgE against tropomyosin, while in 52% of cases, the serum showed reactions with a protein with a molecular weight > 60 kDa. Moreover, sIgE reactivity in the case of proteins with molecular mass corresponding to arginine kinase (Pen m 2, 40 kDa), calcium-binding sarcoplasm binding protein (Lit v 4, 20 kDa), light myosin chain (Lit v 3, 20 kDa), triphosphate isomerase (Cra c8, 27 kDa), troponin C (Cra c 6, 17 kDa), and fatty acid binding protein (15 kDa) was rarely observed (13% in total) [23]. Girffida et al. identified the high molecular weight protein (> 60 Da) as hemocyanin [10]. Recently, Tonomura K et al. described that another clinically important protein with molecular weight of 40 kDa was found and identified as fructose 1,6—bisphosphate aldolase in a case of food dependent, exercise induced anaphylaxis [24]. In 2018, Kimura H et al. identified 43 kDa shrimp allergen as a cause of food dependent, exercise induced anaphylaxis [25].

It is also worth noting that 11 patients were allergic to tropomyosin of house dust mites, which might be associated with cross-allergy with shrimp tropomyosin. However, elevated sIgE to Pen m 1 shrimp tropomyosin was found in only eight patients (Table 2). In our previous studies based on the assessment of the concentrations of sIgE Der p 10 and Pen a 1 in the quantitative ImmunoCap method, the correlation coefficient between these concentrations was as much as 0.987. This suggested that it was economically unjustified to use both determinations, as the results practically coincided [19]. In the present study, the use of the semi-quantitative ImmunoCap ISAC method, characterized by lower sensitivity, resulted in a significantly greater discrepancy between results. It cannot be excluded that the use of other substrates in the ImmunoCap and ImmunoCap ISAC methods may be of influence, as Pen a 1 was extracted from *Penaeus aztecus* and Pen m 1 was extracted from *Peneus Monodon* [26]. At the same time, ten patients were allergic to Bla g 7, German cockroach tropomyosin, which also might be associated with cross-allergy [27].

It is also interesting to analyze other allergen components, which are not related to mites and crustaceans, to which the patient is allergic. It is notable that as many as 16 patients were sensitized to the cat’s main allergen, Fel d 1. 15 patients were sensitized to the main Bermuda grass allergen (Cyn d 1), while 14 patients were allergic to timothy grass (Phl p 1). 13 patients were sensitized to the main allergen of birch-tree (Bet v 1). A high incidence of sensitization to Bet v 1-like proteins (Mal d 1, Cor a 1) was also observed, most likely due to cross-allergy with birch pollen in these patients. This sensitization pattern is probably due to the overlap of the general allergic profile of atopic population in Poland, namely because we are dealing with a selected group of patients allergic to shrimp allergens. In the largest epidemiological study conducted so far in Poland in 2006–2008, involving 20,545 randomly selected citizens, of whom 4,783 had tests done, it was found that the allergy (confirmed by positive skin test results) to 23.4% were allergic to *Dermatophagoides pteronyssinus*, 21.3% were allergic to grass (21.3%), 16.1% were allergic to mugwort, 14.9% were allergic to birch, while 12.3% were allergic to cats. These allergies were confirmed by positive skin test results [28].

Further studies on food allergy developments and alarmins are necessary, due to future possibilities of biological treatment of many different allergic disorders. Currently, most of the studies on potential role of TSLP, IL-25 and IL-33 are on animal models, and the results are promising [29].

In a mouse model, Khodoun et al. found that pro-TH2 cytokines were required to induce food allergy and prevent establishing oral tolerance. Combined treatment with the antagonists of TSLP, IL-25 and IL-33 or with an inhibitor of pro-TH2 cytokine production might be able to suppress established human food allergy [30].

In 2019, the results of phase two of a randomized, placebo-controlled study of anti-IL-33 (etokimab) in peanut allergy were found. In a six-week study, the safety and the ability of a single dose of etokimab to desensitize peanut-allergic adults was evaluated. Participants received either etokimab (n = 15) or blinded placebo (n = 5). Efficacy measurements for active versus placebo participants at the day 15 and day 45 food challenge (a cumulative 275 mg of peanut protein) demonstrated a high, although not long-lasting, effect of etokimab in the group ( 73% vs. 0% to 57% vs. 0%, respectively) [31].

A certain limitation was the relatively small study population, which included a selected group of 37 patients with shrimp allergy and 20 subjects free from allergy symptoms who were the control group. Certainly, a larger population would allow a better interpretation of the study. No oral food challenges with shrimp were performed during the study, and the diagnosis was based on the history and elevated shrimp specific IgE levels. It would also be interesting to determine the relationship between TSLP, IL-25 and IL-33 levels and the time from exposure to the given allergen. However, in this case, polysensitization of patients who were allergic to various allergens, and were exposed to them at different times, was the main hurdle. For this reason, an analysis of alarmin levels in relation to the time passed since consumption of shrimp would be inadequate in this study population. A time of exposure to the level of alarmins study would require a very specific, mono-sensitized population, in which the oral food challenge should be performed. It is an interesting direction for further research on this topic.

## Conclusion

It was found that in patients with an allergy to shrimp, the concentrations of TSLP and IL-25 in the patients’ blood serum were significantly higher than in the control group. No relationship was found between the concentrations of TSLP, IL-25 and IL-33 and the nature and severity of the patients’ clinical symptoms. In the study group of patients with shrimp allergy, sensitization to airborne allergens, mainly house dust mites, grass, birch and cat’s hair, dominated. No relationship was found between the concentrations of TSLP, IL-25 and IL-33 and the concentration of specific IgE in patients or the amount of allergen components the patients were allergic to. Simultaneously, among shrimp-allergic patients with elevated shrimp-specific IgE and a positive history of clinical symptoms, ImmunoCap ISAC allowed sensitization to be detected in only 14 patients. This indicates that ImmunoCap ISAC cannot replace the allergen extract determinations due to the limited amount of shrimp allergen components available in this micro-array test.

## Supplementary Information

**Additional file 1.** Exclusion criteria for the research.

## Data Availability

The dataset supporting the conclusions of this article are included within the article.

## Abbreviations

IgE: Immunoglobulin E
sIgE: Specific Immunoglobulin E
TSLP: Thymic stromal lymphopoietin
IL-33: Interleukin 33
IL -25: Interleukin 25
AD: Atopic dermatitis
HDM: House dust mite
SPT: Skin prick test

## References

[1] Wong CK, Hu S, Cheung PFY, Lam CW (2010). TSLP induces chemotactic and pro-survival effects in eosinophils: implications in allergic inflammation. Am J Respir Cell Mol Biol.

[2] Ziegler SF, Artis D (2010). Sensing the outside world: TSLP regulates barrier immunity. Nat Immunol.

[3] Zhou B, Comeau MR, De Smedt T, Liggitt HD, Dahl ME, Lewis DB (2005). Thymic stromal lymphopoietin as a key initiator of allergic airway inflammation in mice. Nat Immunol.

[4] Headley M, Zhou B, Shih W, Aye T, Comeau M, Ziegler S (2009). TSLP conditions the lung immune environment for the generation of pathogenic innate and antigen-specific adaptive immune responses. J Immunol.

[5] Hui C, Rusta-Sallehy S, Asher I, Heroux D, Denburg J (2014). The effects of thymic stromal lymphopoietin and IL-3 on human eosinophil–basophil lineage commitment: Relevance to atopic sensitization. Immun Inflamm Dis..

[6] Han H, Xu W, Headley MB, Jessup HK, Lee KS, Omori M, Comeau MR, Marshak-Rothstein A, Ziegler SF (2012). Thymic stromal lymphopoietin (TSLP)-mediated dermal inflammation aggravates experimental asthma. Mucosal Immunol.

[7] Imai Y, Yasuda K, Sakaguchi Y, Haneda T, Mizutani H, Yoshimoto T, Nakanishi K, Yamanishi K (2013). Skin-specific expression of IL-33 activates group 2 innate lymphoid cells and elicits atopic dermatitis-like inflammation in mice. Proc Natl Acad Sci U S A.

[8] Han H, Roan F, Johnston LK, Smith DE, Bryce PJ, Ziegler SF (2018). IL-33 promotes gastrointestinal allergy in a TSLP-independent manner. Mucosal Immunol.

[9] Lyons SA, Clausen M, Knulst AC, Ballmer-Weber BK, Fernandez-Rivas M, Barreales L, Bieli C, Dubakiene R, Fernandez-Perez C, Jedrzejczak-Czechowicz M, Kowalski ML, Kralimarkova T, Kummeling I, Mustakov TB, Papadopoulos NG, Popov TA, Xepapadaki P, Welsing PMJ, Potts J, Mills ENC, van Ree R, Burney PGJ, Le TM (2020). Prevalence of food sensitization and food allergy in children across Europe. J Allergy Clin Immunol Pract.

[10] Giuffrida MG, Villalta D, Mistrello G, Amato S, Asero R (2014). Shrimp allergy beyond tropomyosin in italy: clinical relevance of arginine kinase sarcoplasmic calcium binding protein and hemocyanin. Eur Ann Allergy Clin Immunol.

[11] Gour N, Lajoie S, Smole U, White M, Hu D, Goddard P, Huntsman S, Eng C, Mak A, Oh S, Kim JH (2018). Dysregulated invertebrate tropomyosin–dectin-1 interaction confers susceptibility to allergic diseases. Sci Immunol..

[12] Ying S, O'Connor B, Ratoff J, Meng Q, Fang C, Cousins D, Zhang G, Gu S, Gao Z, Shamji B, Edwards MJ, Lee TH, Corrigan CJ (2008). Expression and cellular provenance of thymic stromal lymphopoietin and chemokines in patients with severe asthma and chronic obstructive pulmonary disease. J Immunol.

[13] Tang W (2014). IL-25 and IL-25 receptor expression on eosinophils from subjects with allergic asthma. Int Arch Allergy Immunol.

[14] Nygaard U, Hvid M, Johansen C, Buchner M, Fölster-Holst R, Deleuran M, Vestergaard C (2016). TSLP, IL-31, IL-33 and sST2 are new biomarkers in endophenotypic profiling of adult and childhood atopic dermatitis. J Eur Acad Dermatol Venereol.

[15] Sano Y, Masuda K, Tamagawa-Mineoka R, Matsunaka H, Murakami Y, Yamashita R, Morita E, Katoh N (2013). Thymic stromal lymphopoietin expression is increased in the horny layer of patients with atopic dermatitis. Clin Exp Immunol.

[16] Noti M, Kim BS, Siracusa MC, Rak GD, Kubo M, Moghaddam AE, Sattentau QA, Comeau MR, Spergel JM, Artis D (2014). Exposure to food allergens through inflamed skin promotes intestinal food allergy through the thymic stromal lymphopoietin-basophil axis. J Allergy Clin Immunol.

[17] Yang AC, Arruda LK, Santos AB, Barbosa MC, Chapman MD, Galvão CE, Kalil J, Morato-Castro FF (2010). Measurement of IgE antibodies to shrimp tropomyosin is superior to skin prick testing with commercial extract and measurement of IgE to shrimp for predicting clinically relevant allergic reactions after shrimp ingestion. J Allergy Clin Immunol.

[18] Rosenfield L, Tsoulis MW, Milio K, Schnittke M, Kim H (2017). High rate of house dust mite sensitization in a shrimp allergic southern Ontario population. Allergy Asthma Clin Immunol.

[19] Ukleja-Sokołowska N, Gawrońska-Ukleja E, Lis K, Żbikowska-Gotz M, Adamczak R, Sokołowski Ł, Bartuzi Z (2020). Shrimp sensitization in house dust mite allergic patients. Int J Immunopathol Pharmacol.

[20] Reese G, Schicktanz S, Lauer I, Randow S, Luttkopf D, Vogel L, Lehrer SB, Vieths S (2006). Structural, immunological and functional properties of natural recombinant Pen a 1, the major allergen of Brown Shrimp. Penaeus aztecus Clin Exp Allergy.

[21] Moreno FJ (2007). Gastrointestinal digestion of food allergens: effect on their allergenicity. Biomed Pharmacother.

[22] Ukleja-Sokołowska N, Lis K, Żbikowska-Gotz M, Adamczak R, Kuźmiński A, Bartuzi Z (2021). Clinical utility of immunological methods based on the singleplex and multiplex ImmunoCap systems for diagnosis of shrimp allergy. J Int Med Res.

[23] Asero R, Mistrello G, Amato S (2012). Shrimp allergy in Italian adults; a multi center study showing a high prevalence of sensitivity to novel high molecular weight allergens. Int Arch Allergy Immunol.

[24] Tonomura K, Fujimoto R, Okuda Y, Iba N, Sakamoto S, Kosugi E, Kishida H, Matsuo H, Kataoka Y (2019). A case of food-dependent exercise-induced anaphylaxis by shrimp: fructose 1, 6- bisphosphate aldolase is supposed as causative component despite negative allergen-specific IGE test (IMMUNOCAP^®^). Arerugi.

[25] Kimura H, Inami M, Hamaguchi Y, Takehara K, Akimoto S, Yokooji T, Matsuo H, Matsushita T (2018). Food-dependent exercise-induced anaphylaxis due to shrimp associated with 43 kDa, a new antigen. J Dermatol.

[26] Phadia Allergen Information http://www.phadia.com/en/Products/Allergy-testing-products/ImmunoCAP-Allergen-Information/Food-of-Animal-Origin/Allergen-Components/rPen-a-1-Tropomyosin-Shrimp/. Accessed 30 Apr 2020.

[27] Mohamad Yadzir ZH, Misnan R, Abdullah N, Bakhtiar F, Leecyous B, Murad S (2014). Component-resolved diagnosis (CRD): Is it worth it? frequency and differentiation in rhinitis patients with mite reactivity. Iran J Allergy Asthma Immunol.

[28] ECAP results. http://ecap.pl/pdf/ECAP_wyniki_pl.pdf. Accessed 30 Apr 2020.

[29] Ukleja-Sokolowska N, Bartuzi Z, IL 25, IL 33 and TSLP in atopic diseases—current state of knowledge. Alergia Astma Immunologia. 2020;25(2). http://www.alergia-astma-immunologia.pl/2020_25_2/AAI_02_2020_1383_ukleja.pdf.

[30] Khodoun MV, Tomar S, Tocker JE, Wang YH, Finkelman FD (2018). Prevention of food allergy development and suppression of established food allergy by neutralization of thymic stromal lymphopoietin, IL-25, and IL-33. J Allergy Clin Immunol.

[31] Chinthrajah S, Cao S, Liu C, Lyu SC, Sindher SB, Long A, Sampath V, Petroni D, Londei M, Nadeau KC (2019). Phase 2a randomized, placebo-controlled study of anti-IL-33 in peanut allergy. JCI Insight.

