# Supplementary material for: Assessment of TSLP, IL 25 and IL 33 in patients with shrimp allergy
Source: Allergy Asthma Clin Immunol. 2021 Jul 23;17:76. doi: 10.1186/s13223-021-00576-9 (PMC8299623; doi:10.1186/s13223-021-00576-9)
Supplement: Supplementary file 1 — Additional file 1. Exclusion criteria for the research. [file 13223_2021_576_MOESM1_ESM.docx]

| Age <18 year old |
| --- |
| Mental disorders that would not allow to give informed consent |
| Uncooperating patient |
| Pregnant or breastfeeding women |
| Any acute condition |
| Uncontrolled bronchial asthma |
| Past or undergoing oncological treatment |
| Rheumatological disorders |
| Autoimmunological disorders |
| Undergoing viral or bacterial infection |
| Medication – antihistamines, systemic steroids, anti-depressants |
| Immunotherapy for inhaled allergens or Hymenoptera venom – past or undergoing |

Additional file 1: Exclusion criteria for the research
